# Global Perspective of the Vitamin D Status of African-Caribbean Populations: A Systematic Review and Meta-analysis

**DOI:** 10.1038/s41430-021-00980-9

**Published:** 2021-07-19

**Authors:** Rebecca M. Vearing, Kathryn H. Hart, Andrea L. Darling, Yasmine Probst, Aminat S. Olayinka, Jeewaka Mendis, Helena Ribeiro, Siddhartha Thakur, Marcela Mendes, Karen Charlton, Susan A. Lanham-New

**Affiliations:** 1 School of Biosciences and Medicine, Faculty of Health and Medical Sciences, University of Surrey, Guildford, UK; 2 School of Medicine, Faculty of Science Medicine and Health, University of Wollongong, Wollongong, NSW Australia; 3 Illawarra Health and Medical Research Institute, Wollongong, NSW Australia; 4 Surrey Clinical Trials Unit, University of Surrey, Guildford, UK; 5 School of Public Health, University of São Paulo, São Paulo, USA; 6 College of Veterinary Medicine, North Carolina State University, Raleigh, NC USA; 7 School of Nutrition, Faculty of Health Sciences, University of Brasilia, Brasilia, Brazil

**Keywords:** Calcium and vitamin D, Bone

## Abstract

**Background/Objectives:**

Vitamin D deficiency remains a global public health issue, particularly in minority ethnic groups. This review investigates the vitamin D status (as measured by 25(OH)D and dietary intake) of the African-Caribbean population globally.

**Subjects/Methods:**

A systematic review was conducted by searching key databases (PUBMED, Web of Science, Scopus) from inception until October 2019. Search terms included ‘Vitamin D status’ and ‘African-Caribbean’. A random effects and fixed effects meta-analysis was performed by combining means and standard error of the mean.

**Result:**

The search yielded 19 papers that included *n* = 5670 African-Caribbean participants from six countries. A meta-analysis found this population to have sufficient (>50 nmol/L) 25(OH)D levels at 67.8 nmol/L, 95% CI (57.9, 7.6) but poor dietary intake of vitamin D at only 3.0 µg/day, 95% CI (1.67,4.31). For those living at low latitudes ‘insufficient’ (as defined by study authors) 25(OH)D levels were found only in participants with type 2 diabetes and in those undergoing haemodialysis. Suboptimal dietary vitamin D intake (according to the UK recommended nutrient intake of 10 µg/day) was reported in all studies at high latitudes. Studies at lower latitudes, with lower recommended dietary intakes (Caribbean recommended dietary intake: 2.5 µg/day) found ‘sufficient’ intake in two out of three studies.

**Conclusions:**

25(OH)D sufficiency was found in African-Caribbean populations at lower latitudes. However, at higher latitudes, 25(OH)D deficiency and low dietary vitamin D intake was prevalent.

## Introduction

The majority of vitamin D is derived from sunlight exposure, rather than food [1, 2]. When ultraviolet-B (UVB) radiation (wave length 290–315 nm) comes into contact with the skin, 7-dehydrocholesterol (provitamin D_3_) converts to vitamin D_3_ (cholecalciferol) [3]. In total, 80–100% of vitamin D is synthesised in this way, with the remainder coming from dietary intake of food and/or supplements [4–7]. Vitamin D is naturally present in a limited number of foods including animal sources (cholecalciferol) such as oily fish, red meat, dairy and eggs and plant sources (ergocalciferol) such as UVB exposed mushrooms [3]. Vitamin D from food sources is absorbed and converted to 25-hydroxyvitamin D [25(OH)D] in the liver, along with vitamin D_3_ from sunlight synthesis. 25(OH)D is the major circulating form of vitamin D. It can be used as a marker of vitamin D status, as it reflects the synthesis from sun exposure as well as from dietary intake [7]. In the kidneys, 25(OH)D is metabolised to the biologically active form of vitamin D, 1,25[OH]_2_D (calcitriol) [8]. The main role of this active form is the maintenance of calcium homeostasis, and therefore, musculoskeletal health [2, 9]. In addition, most cells in the body have a vitamin D receptor, meaning that vitamin D influences many biological pathways, and vitamin D deficiency is associated with disease states including certain cancers, type 2 diabetes mellitus (T2DM), multiple sclerosis, immune response and cardiovascular disease [2, 8, 10–14]. In this review, vitamin D status will refer to 25(OH)D, which is mainly from cutaneous synthesis. Dietary intake includes vitamin D sourced from both ergocalciferol and cholecalciferol.

Vitamin D synthesis is multifactorial, with both environmental and personal factors impacting on an individual’s ability to synthesise vitamin D cutaneously, and to consume it through their diet. Low UVB exposure, which may relate to latitude, season, pollution or urbanisation, are key factors in vitamin D synthesis [7, 15, 16] while personal characteristics, including ethnicity, skin type, age and sun exposure habits, may lead to higher or lower vitamin D synthesis [11]. Vitamin D deficiency may lead to rickets in children, or osteomalacia and osteoporosis in adults, as well as other non-bone related conditions [17].

Vitamin D deficiency remains a global public health issue, particularly in minority ethnic groups [18]. There is a lack of research investigating the vitamin D status of the African-Caribbean (AfC) population globally. This population refers to those with African ancestry who migrated via the Caribbean, or those native to the Caribbean with African ancestry [19]. This population has darkly pigmented skin, which reduces the capacity to synthesise vitamin D. This is because increased melanin content acts as a natural ‘sunscreen’ [11, 20].

AfC populations are known to have low levels of vitamin D deficiency [17, 21]. However, research to date has mostly been undertaken in countries with low latitudes and high year-round sun exposure. Migration away from the equator to higher latitudes, and thus reduced sun exposure, has had consequences for vitamin D concentration for populations with darker skin types [3, 11]. Further, the ability to synthesise vitamin D from sunlight is restricted to the summer months in countries higher than 37°N [22]. Vitamin D status in the winter months in those countries is reliant solely on dietary or supplemental intakes, which is often inadequate to meet requirements. Thus, it is likely that AfC people living at higher latitudes have a poorer vitamin D status than those living closer to the equator. This hypothesis is partially supported by a review by Wacker et al. [11] that reported a significant inverse relationship between mean circulating 25(OH)D levels and latitude in European young adults. Whether these findings would translate to an AfC population is yet unknown.

This systematic review and meta-analysis characterises the current vitamin D status of AfC populations globally, residing at different latitudes and geographical regions, in order to identify clinical and public health need, as well as areas of future research. In particular, this review provides key data on vitamin D concentration in a variety of African-Caribbean populations, which will inform the development of policy as well as clinical decision making, globally.

## Methods

### Literature search

A search for relevant literature was undertaken in October 2019 using the PUBMED, Web of Science and Scopus scientific databases. The following search including MeSH terms were used: (Vitamin D intake OR dietary vitamin D OR vitamin D supplement OR vitamin D consumption OR Vitamin D status OR vitamin D level OR 25(OH)D OR 25-hydroxy*) AND (African-Caribbean OR afro-Caribbean OR African OR Caribbean). The literature search was not limited to a time period but included only human studies that were published in the English language (See Supplementary File Table 1 for the full search criteria).

### Eligibility criteria for inclusion and data extraction

One author (RMV) screened the titles and abstracts of potential papers and then screened the full text papers for final inclusion. Additionally, a random sample (10%) of both the abstracts and full text papers were cross-checked by a second author (ASO). Any discrepancies were deferred to a third party where required (ALD, KHH, SLN).

Papers that assessed vitamin D status as a primary outcome, either through dietary vitamin D intake or serum 25(OH)D concentration, in an AfC population were included. Any human studies with a population described or self-identified as “African-Caribbean/Afro-Caribbean/Black Caribbean” were included, as well as studies with Caribbean populations of African ancestry. Those papers which did not specifically define an AfC population, or described their population as ‘African-American’ or ‘African’ were excluded [19]. All age and gender groups were included, including infants and pregnant women, as well as those with diagnosed disease states. Studies at low latitudes (0–37° North and South) and high latitudes (37–90° North and South) were included. Reviews and conference abstracts were excluded.

At the first stage of screening, populations described as ‘black’ were included. At full text screening if a ‘black’ population referred to anything other than an AfC population, the paper was excluded. Papers with populations described as ‘black’, which included AfCs, were excluded if specific data for the primary outcome had not been presented for the AfC participants alone. Study authors were contacted for further information regarding ethnicity, as required.

Information was extracted from the included studies to summarise the author and publication year, study design, latitude, population, primary outcomes and secondary outcomes. The actual data for relevant outcomes were also extracted. We followed the Preferred Reporting Items for Systematic Reviews and Meta-Analyses (PRISMA) [23] guidelines (See PRISMA checklist). The review was registered with PROSPERO number: CRD42019158108, https://www.crd.york.ac.uk/PROSPERO/.

### Data and statistical analysis

A meta-analysis was performed, using the ‘rmeta’ package within R Studio [24], combining means and standard error of the mean (SEM), to obtain a pooled estimate. Studies were included in the meta-analysis if they provided adequate data in terms of means and SEM for vitamin D 25(OH)D concentration and/or vitamin D dietary intake. If this was not provided, authors were contacted to request further data. As required, SEM was calculated by dividing the standard deviation (SD) of the mean by the square root of the sample size. If only median and IQR were presented, then they were converted to mean and SD [25]. If sufficient data could not be obtained, the study was excluded from the meta-analysis [26, 27]. Random and fixed effects models were used, and heterogeneity was assessed (*p* < 0.05 was considered as statistically significant heterogeneity). A sensitivity analysis was conducted by removing each study from the analysis in turn and inspecting effect size. A Pearson’s correlation was conducted to assess the relationship between latitude and 25(OH)D concentration.

Methods for measuring vitamin D status, as well as the cut-offs used to define sufficiency and deficiency, differ in the literature. This review categorised vitamin D cut-offs as: vitamin D deficiency (<25 nmol/L) [22], insufficiency (>25–50 nmol/L) [28] and sufficiency (>50 nmol/L) [28] (Supplementary File and Table 2). Recommended vitamin D dietary intakes varied between the countries of the papers included in this review, with adults in the United Kingdom (UK) being recommended to consume 10 µg/day of vitamin D [22] while those in the Caribbean islands advised to achieve 2.5 µg/day [29] (Supplementary File and Table 3). Therefore, dietary intake was compared to the recommendations for the relevant country the study was conducted in.

Data on 25(OH)D concentrations were analysed using units of nmol/L, and dietary vitamin D intake using µg/day. Where authors reported concentrations in ng/mL, values were converted to nmol/L using a multiple of 2.5 [11]. Where authors reported intake in IU/day, values were multiplied by 0.025 to convert units to µg/day [22].

### Quality analysis

Quality rating of all papers was performed by one author (RMV). The Newcastle Ottawa Scale [30] was used to assess the quality of design and conduct of the case–control studies included in this review. A modified scale adapted by Herzog et al. [31] was used to quality rate cross-sectional and cohort studies [32].

## Results

### Systematic review

**Fig. 1:**
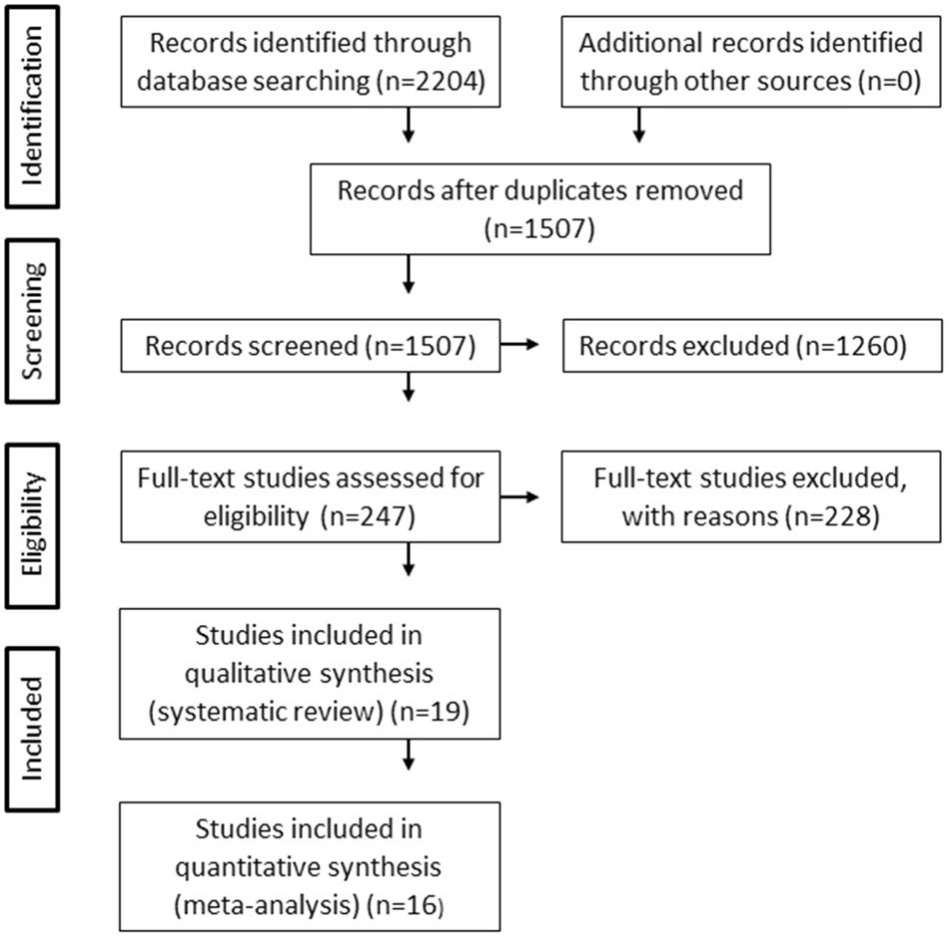
PRISMA flow diagram showing study selectionAQ5 process [23]. PRISMA flow diagram summarising the results of the search and selection processes.

The initial search resulted in 2204 papers. After duplicates were removed, 1507 papers were screened by title and abstract. Finally, the full text of 247 papers were screened [23] (See Fig. 1 for PRISMA flow diagram). For reasons for exclusion, see the Supplementary File and Table 4.

Nineteen observational studies, exploring the vitamin D status or dietary intake of the AfC population across six countries with different latitudes were included (*n* = 5670 participants) (see Tables 1 and 2). Jackson et al. [33] reported on both 25(OH)D concentration and dietary intake, so is included in both the 25(OH)D and dietary intake qualitative analyses (see Supplementary File and Table 5). The studies varied in terms of quality, with most studies being considered fair to good quality (Supplementary File and Tables 6 and 7). The included papers were all published in the previous 15 years, from 2005 to 2019.

**Table 1:** Summary table for 25(OH)D concentrations of African-Caribbean populations.

Study design	Reference and study design	Country/latitude	Participant characteristics	Mean age (years)	Primary outcomes	Vitamin D deficiency cut-off (nmol/L)^b^	Vitamin D insufficiency cut-off (nmol/L)^b^	Vitamin D sufficiency cut-off (nmol/L)^b^	Results/findings
Cross-sectional	Barbour et al. [33]	Caribbean island of Tobago 11°N	*n* = 496 community-dwelling AfC men	77.5 ± 5.1	25(OH)D (LC-MS/MS)	<50	50–<75	≥75	AfC had higher mean 25(OH)D than WE (86.61 ± 24.21 vs. 68.89 ± 20.72 nmol/L^b^, *p* < 0.001)
Cross-sectional	Chiang et al. [9]	Chicago, IL (latitude: 41°N); Kingston, Jamaica (17°N); Kumasi, Ghana (6°N); Victoria, Seychelles (4°S); and Cape Town, South Africa (34°S)	*n* = 2242 healthy adults (46% men) from the VIDA study, *n* = 448 Jamaicans (AfC), *n* = 1794 others with African ancestry	34.4 ± 6.1	25(OH)D (LC-MS/MS)	–	<50	≥50	25(OH)D concentration as reported in Durazo-Arvizu et al.
Cross-sectional	Crew et al. [42] Additional unpublished data provided by author^a^	Brooklyn, New York 41°N	*n* = 114 healthy women, *n* = 21 AfC, 28 white, 47 AA, 18 Hispanic	50 ± 5.8	25(OH)D (DiaSorin RD)	<50	50–^~^75	≥75	Mean 25(OH)D of AfC 56.66 ± 20.97 nmol/L. Higher 25(OH)D associated with white ethnicity
Cross-sectional	Durazo-Arvizu et al. [34]	Chicago, IL (latitude: 41°N); Kingston, Jamaica (17°N); Kumasi, Ghana (6°N); Victoria, Seychelles (4°S); and Cape Town, South Africa (34°S)	*n* = 459 (50% female) Jamaicans (AfC) and *n* = 1990 others	34.7 ± 0.99	25(OH)D (LC-MS/MS)	<30	30–50	≥50	Mean 25(OH)D of AfC: 72.13 ± 17.72 nmol/L. Negative correlation between 25(OH)D and latitudinal distance from the equator. No AfC were at risk of deficiency, 90% were sufficient
Cross-sectional	Ford et al. [43]	Birmingham, United Kingdom, 52°N	*n* = 831 adults (500 women), *n* = 125 AfC, *n* = 705 others	53.2 ± 0.66	25(OH)D (chemiluminescence immunoassay)	<25	–	–	Mean 25(OH)D of AfC: 39.94 ± 21.77 nmol/L^b^. 1 in 4 AfC were vitamin D deficient at the end of summer
Cross-sectional	Foucan et al. [35]	Caribbean island of Guadeloupe, 16°N	*n* = 152 AfC adults (46.1% women) undergoing haemodialysis	61 ± 14	25(OH)D (direct Radioimmunoassay)	–	<75	≥75	Weighted mean 25(OH)D concentration for AfC 70.29 ± 26.51 nmol/L^b^. 60% were vitamin D insufficient
Observational	Hwang et al. [21]	Caribbean Island of Tobago, 11°N	*n* = 574 healthy adult AfC men from the TBHS	59.1 ± 10.5	25(OH)D (LC-MS/MS) 1,25-dihydroxyvitamin D (RIA)	<50	50–^~^75	>75	Mean 25(OH)D concentration 84.61 ± 22.76 nmol/L^b^. Vitamin D deficiency was found in 3.8% and vitamin D insufficiency was found in 33.1% of participants
Case control	Jackson et al. [36]	Caribbean island of Jamaica, 18°N	*n* = 337 AfC participants, newly diagnosed with PCa (146 cases, 191 controls)	62.3 ± 10.5 (controls), 67.6 ± 7.8 (cases)	25(OH)D (UPLC-MS/MS)	<50	50–75	>75	Weighted mean 25(OH)D 82.19 ± 79.87 nmol/L^b^. 12.4% were vitamin D deficient
Cross sectional	McGhie et al. [37]	Caribbean island of Jamaica, 18°N	*n* = 75 AfC patients with SLE (92% female)	41.5 ± 14.3	25(OH)D (enzyme-linked immunoassay)	<20	20–75	>75	Mean 25(OH)D 76.13 ± 25.71 nmol/L^b^. 44% had vitamin D sufficiency, 56% were deficient or insufficient
Cohort	Miljkovic et al. [17]	Caribbean island of Tobago 11°N	*n* = 424 healthy AfC men	72 ± 5.8	25(OH)D (LC-MS/MS)	<20	20–75	>75	Mean 25(OH)D 87.61 ± 22.21 nmol/L^b^. 2.8% were deficient and 24% were insufficient
Cross-sectional	Naqvi et al. [38]	Caribbean island of Guatemala, 15°N	*n* = 86 healthy adolescents, from local ethnic groups: *n* = 43 Garifunas (AfC) and 43 Kekchi Mayans	13.5 ± 1.6 years	25(OH)D (CLIA)	<20	20–75	>75	Mean 25(OH)D of AfC 74.38 ± 19.82 nmol/L^b^. 5% of AfC were deficient, 43% had adequate levels
Cross-sectional	Patel et al. [27]	Birmingham, United Kingdom, 52° N	*n* = 782 AfC adults (51% male), and *n* = 1112 SA (56% male)	61.7 (60.9–62.5) (AfC)	25(OH)D (mass spectrometer)	<15 (severe), 15–30 (deficiency)	30–50	>50	14.1% (10.3,17.9) of AfC were severely deficient, 40.6% (35.4, 45.9) deficient, 33.6% (28.5, 38.7) insufficient and 13.2% (9.6, 16.9) adequate
Cross-sectional	Rezai et al. [44] Additional unpublished data provided by author^a^	Manchester, United Kingdom, 53°N	*n* = 67 community-dwelling AfC men and *n* = 68 SA, and *n* = 63 Eu	55 ± 10 (AfC)	25(OH)D (LC-MS/MS)	<50	–	–	Mean 25(OH)D AfC 28 ± 2 nmol/L. AfC had 14 nmol/L and SA had 21 nmol/L lower mean 25(OH)D than EU (*p* < 0.001)
Cross-sectional	Velayoudom-Cephise et al. [39]	Caribbean island Guadeloupe, 16°N	*n* = 201 AfC and *n* = 76 Guadeloupeans adults with T2DM	64 ± 11	25(OH)D (direct immunoassay)	<50	<75	>75	Mean 25(OH)D AfC concentration 54.16 ± 17.24 nmol/L^b^.

*AA* African-American, *AfC* African-Caribbean, *CI* confidence intervals, *CLIA* chemiluminescent immunoassay, *Eu* European, *LC-MS/MS* liquid Chromatography and Tandem Mass Spectrometry, *PCa* prostate cancer, *RIA* Radioimmunoassay, *SA* South Asian, *SLE* systemic lupus erythematosus, *UPLC-MS/MS* ultraperformance liquid chromatography/tandem mass spectrometry, *VIDA* Vitamin D Ancillary, *WE* White Europeans, *25(OH)D* serum 25-hydroxyvitamin D.

^a^Unpublished data provided by author.

^b^Note: vitamin D concentrations converted to nmol/L, where authors originally published results in ng/ml or µg/L. Values were rounded to whole numbers for vitamin D cut-offs.

**Table 2:** Summary table for vitamin D dietary intake of African-Caribbean populations.

Reference and study design	Study design	Country/latitude	Participants characteristics	Mean age (years)	Primary outcomes	Vitamin D supplement use	Dietary intake recommendation for country	Results/findings
Castaneda-Gameros et al. [46] Additional unpublished data provided by author^a^	Cross sectional	Birmingham, United Kingdom, 52°N	*n* = 21 AfC, 20 Indian, 10 African, 8 Arab, 7 Pakistani, 5 Bangladeshi, 5 Irish elderly migrant women	70.5 ± 7.6	Dietary intake (24-h recall and dietary interview)	Some	UK RNI 10 µg/day	Mean vitamin D intake AfC 9.63 ± 7.5 µg/day (range 1.23–28.38 µg/day). Vitamin D intake for entire population was significantly lower (*p* = 0.02) than RNI (10 µg/day)
Donin et al. [47]	Cross-sectional	London, 52° N, Birmingham 52° N and Leicester, 53° N, United Kingdom	*n* = 2209 children from the CHASE study: *n* = 560 AfC, *n* = 558 SA, *n* = 543 EU	9.9 (9.2, 10.7)	Dietary intake (24-h recall)	Did not report	UK RNI 10 µg/day	Mean vitamin D intake AfC 1.7 ± 1.2 µg/day (95% CI 1.6,1.8). Mean difference in Vitamin D intake (µg/day) between AfC and EU: −12.1 (95% CI −21.5, −1.6, *p* = 0.03).
Jackson et al. [36] Additional unpublished data provided by author^a^	Case–control	Caribbean island of Jamaica, 17°N	*n* = 337 AfC men newly diagnosed with PCa, *n* = 146 participants and 191 controls	62.3 ± 10.5 (controls), 67.6 ± 7.8 (cases)	Dietary intake (FFQ)	29.5% (controls), 23.5% (cases)	Caribbean RDA 2.5 µg/day	Weighted mean vitamin D intake 3.66 ± 2.95 µg/day^b^
Kramer et al. [41]	Cross-sectional	Chicago, IL (latitude: 41°N); Kingston, Jamaica (AfC) (17°N); Kumasi, Ghana (6°N); and Cape Town, South Africa (34°S)	*n* = 396 (47.5% males) healthy Jamaicans (AfC), *n* = 1458 others	34.7 ± 6.2	Dietary intake (2 × 24-h recall)	Did not report	Caribbean RDA 2.5 µg/day	Weighted mean vitamin D intake 2.9 ± 3.4 µg/day
Pakseresht et al. [45]	Cross sectional	Caribbean island of Barbados, 13°N	*n* = 49 AfC adults with breast of prostate cancer (53% female)	60 ± 13	Dietary intake (QFFQ and 4-day food diary)	Did not report	Caribbean RDA 2.5 µg/day	Mean Vitamin D intake from QFFQ: 1.4 ± 1.3 µg/day, from food diary 1.0 ± 1.3 µg/day.
Rees et al. [26]	Cross sectional	London, United Kingdom, 52° N	*n* = 165 post-partum mothers: *n* = 40 AfC, 63 WE, 36 Africans, 26 Asians	Mean age 28.8 ± 5.6	Dietary intake (7-day diet diary)	Those taking supplements excluded from study	UK RNI 10 µg/day	Mean vitamin D intake AfC 3.18 µg/day (ANOVA F statistic 4.1).

*AfC* African-Caribbeans, *CHASE* Child Heart Health Study, *Eu* European, *FFQ* Food Frequency Questionnaire, *PCa* prostate cancer, *QFFQ* Quantitative Food Frequency Questionnaire, *RDI* recommended dietary intake, *RNI* reference nutrient intake, *SA* South Asian, *WE* White Europeans.

^a^Unpublished data provided by author.

^b^Note: vitamin D dietary intake have been converted to µg/day, where authors originally published results in IU/day.

### 25(OH)D concentration of African-Caribbeans living in low latitudes (0–37° North and South)

Ten of the included papers explored the 25(OH)D of an AfC population living at low latitudes (Caribbean islands), with high year-round sun exposure (*n* = 3209 participants) [9, 17, 21, 33–39]. All of these studies reported AfC participants to have ‘sufficient’ vitamin D levels according to assigned cut-offs, as stated by the study authors. As an exception, Velayoudom-Cephise et al. [39] described their AfC participants with T2DM as having ‘insufficient’ vitamin D levels, with a mean 25(OH)D concentration of 54.16 ± 17.22 nmol/L. The study found ‘deficiency’ (<50 nmol/L) in 42.6% of the population, despite sunny climates [39]. Similarly, Foucan et al. [36] found ‘insufficient’ (<75 nmol/L) 25(OH)D concentration (mean 70.29 ± 26.51 nmol/L) in their population of haemodialysis patients.

AfC populations living close to the equator had higher 25(OH)D concentrations when compared to their non AfC counterparts [34, 38, 39]. For example, Barbour et al. [34] found AfCs living in the Caribbean had significantly higher 25(OH)D concentrations compared to those with White European ancestry living in the US (86.61 ± 24.21 vs. 68.89 ± 20.72 nmol/L, *p* < 0.001). Similarly, Foucan et al. [36] found that AfC dwelling close to the equator had lower rates of vitamin D ‘insufficiency’ when compared to a previous study with African Americans (AA) living in United States of America (US) (60% vs 80% respectively, *p* < 0.001) [40]. Likewise, a study by Naqvi et al. [38] found a higher concentration of 25(OH)D in AfC compared to the Indigenous Mayan population, living in the Caribbean islands (74.38 ± 19.82 nmol/L vs. 64.47 ± 14.55 nmol/L).

Three of these studies reported on the results of the Vitamin D Ancillary Study (VIDA) [9, 35, 41]. This study compared participants of African ancestry living at different latitudes and found, according to author defined cut-offs, 90% of AfC participants living in Jamaica (17°N) to have ‘sufficient’ vitamin D levels, and none to be deficient [9, 35, 41]. Interestingly, they also found, a negative correlation between latitudinal distance from the equator and 25(OH)D concentrations, with those of African ancestry living in Jamaica (17°N) having a higher vitamin D concentration when compared to those with African ancestry living in the US (41°N) (72.13 ± 17.72 nmol/L vs 42.93 ± 19.96 nmol/L) [9, 35, 41].

### 25(OH)D concentration of African-Caribbeans living in mid to high latitudes (37–90° North and South)

Four studies explored the vitamin D status of AfC populations living at higher latitudes (UK and US) (*n* = 995 participants) [27, 42–44]. One study was carried out in the US (41°N) [42], whilst the other three were in the UK (52–53°N) [27, 43, 44]. In contrast to AfC populations living at low latitudes, these studies found the mean 25(OH)D concentration of their participants to be ‘insufficient’ to ‘deficient’ according to differing author assigned cut-offs, ranging from 28.0 ± 2.0 to 56.66 ± 20.97 nmol/L [27, 42–44], whilst according to our pre-determined cut-offs, the participants were vitamin D insufficient [27, 43, 44] or sufficient [42].

Of the three studies that compared an AfC population to a White Europeans (WE) population, living in the same location, found higher concentrations of 25(OH)D in the WE population [42–44]. Crew et al. [42] found that higher levels of 25(OH)D were associated with WE ethnicity. Ford et al. [43] reported that WE had the highest mean levels of 25(OH)D, followed by AfCs and then South Asians. Of note, this study also showed that one in every four AfCs living in the UK (52° N) were vitamin D ‘deficient’, according to the author’s definition (<25 nmol/L) after summer [43]. Likewise, Rezai et al. [44] reported a deficient (<50 nmol/L) mean 25(OH)D of 28 ± 2 nmol/ in their AfC sample, which was a 14 nmol/L lower than the mean concentration of their WE counterparts (*p* < 0.001). Patel et al. [27] found that only 15.4% of AfC participants living in the UK had adequate vitamin D levels (defined as >50 nmol/L).

**Fig. 2:**
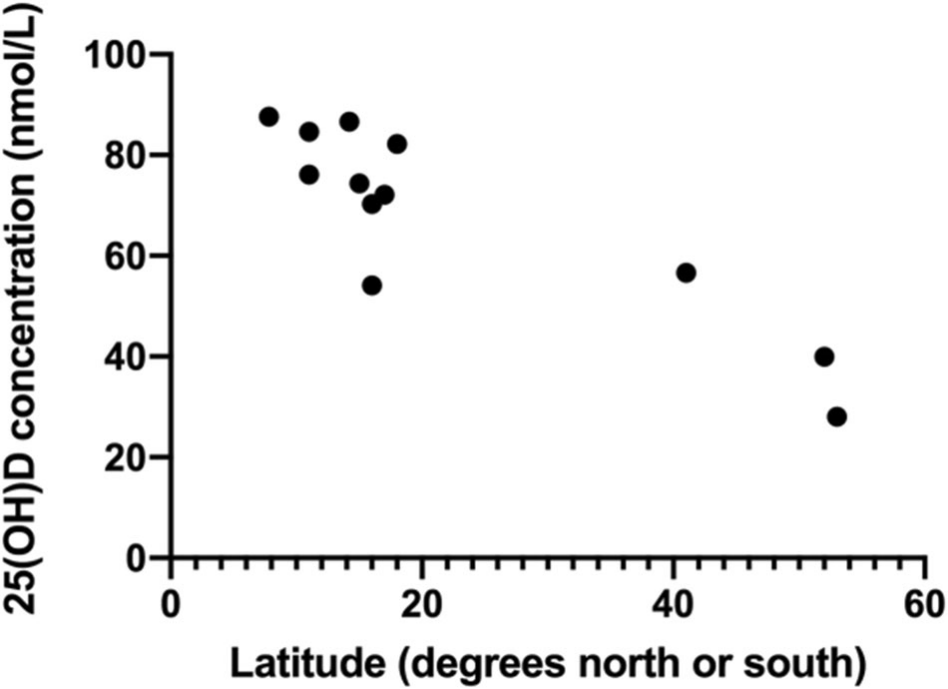
Relationship between vitamin concentration [25(OH)D] and latitude in the African-Caribbean population. Note: 95% confidence interval: −1.210, −0.577, *p* < 0.0001. Mean 25(OH)D concentration of 67.8 nmol/L, 95% CI (57.9, 77.6) from the 12 papers (*n* = 2974, globally) included in the meta-analysis on 25(OH)D concentration [17, 21, 33–39, 42–44]. Latitude reported by author or estimated. Additional unpublished data was provided by some authors.

We found a strong inverse association (Pearson’s correlation) between 25(OH)D status and distance from the equator (*r* = −0.894, *p* < 0.0001) across the 12 papers [17, 21, 33–39, 42–44] included in the sub sample that measured 25(OH)D at different latitudes (Fig. 2).

### Vitamin D dietary intake at low latitudes 0–37° North and South

Three studies measured vitamin D dietary intake in AfC populations living close to the equator (Caribbean islands) (*n* = 782 participants) [33, 41, 45]. The studies varied in terms of the tools used to measure intake, including food frequency questionnaire [33, 45], a 24 h food recall [41] and a 4-day food diary [45]. The Caribbean islands have a low recommended dietary allowance (RDA) for vitamin D of 2.5 µg/day [29]. Although intakes were low, ranging from 1.0 to 3.7 µg/day of vitamin D, two studies had ‘sufficient’ mean intakes when compared to the local RDA for this population [33, 41]. However, in another study, the mean intake of vitamin D for Caribbean island participants with breast or prostate cancer did not meet recommendations [45]. This may be partly explained by the fact that these research participants had cancer, so may not have had normal food intake.

### Vitamin D dietary intake at high latitudes 37–90° North and South

Three studies measured dietary intake of vitamin D at high latitudes (all in the UK) (*n* = 621 participants) [26, 46, 47]. All studies included used a 24-h recall to assess dietary intake [26, 47], whilst in addition, Castaneda-Gameros et al.[46] also used a dietary review. The UK recommended nutrient intake (RNI) for vitamin D is 10 µg/day [22]. Low vitamin D dietary intake was seen in all the studies, ranging from 1.7 to 9.6 µg/day. A study of *n* = 40 post-partum mothers by Rees et al. [26], found that AfC women living in the UK, although having low intakes of vitamin D, still reported slightly higher mean intakes than those of WE or Asian ancestry. Conversely, in another study, inadequate dietary vitamin D was seen in AfC children (1.71 µg/day), with intakes lower than that of their WE counterparts (1.9 µg/day), but higher than those of South Asian children (1.4 µg/day) [47]. A small study by Castaneda-Gameros et al. in older migrant women of mixed ethnicity found vitamin D to be a nutrient of concern, with a median intake of 2.6 µg/day (IQR 0.7–11.4), significantly lower than the UK RNI (*p* = 0.02). However, in a sub sample (*n* = 21) of AfC women, in the same study, unpublished data provided by the authors showed a mean intake of 9.6 µg/day vitamin D, which, included participants who used vitamin D containing supplements [46].

### Meta-analysis

Sixteen studies were included in the meta-analysis, which involved analysis of 25(OH)D concentration [17, 21, 33–39, 42–44] and vitamin D dietary intake [33, 41, 45–47] of AfC populations living at different latitudes. Jackson et al. [33] reported on both 25(OH)D and dietary intake. The remaining three studies were excluded due to insufficient data [26, 27] or reporting on the same data as another author, in which case the study published first was used [9].

### 25(OH)D concentration

Twelve studies reported 25(OH)D concentration (*n* = 2974 participants) [17, 21, 33–39, 42–44]. The pooled effect size for 25(OH)D concentration in AfC populations was a mean_(random)_ of 67.8 nmol/L, 95% CI (57.9, 77.6), with statistically significant heterogeneity (*P*_(heterogeneity)_ < 0.001). A pooled mean_(fixed)_ of 73.5 nmol/L, 95% CI (72.7, 74.3) with statistically significant heterogeneity (*P*_(heterogeneity)_ < 0.001) was found in a fixed effects model.

**Fig. 3:**
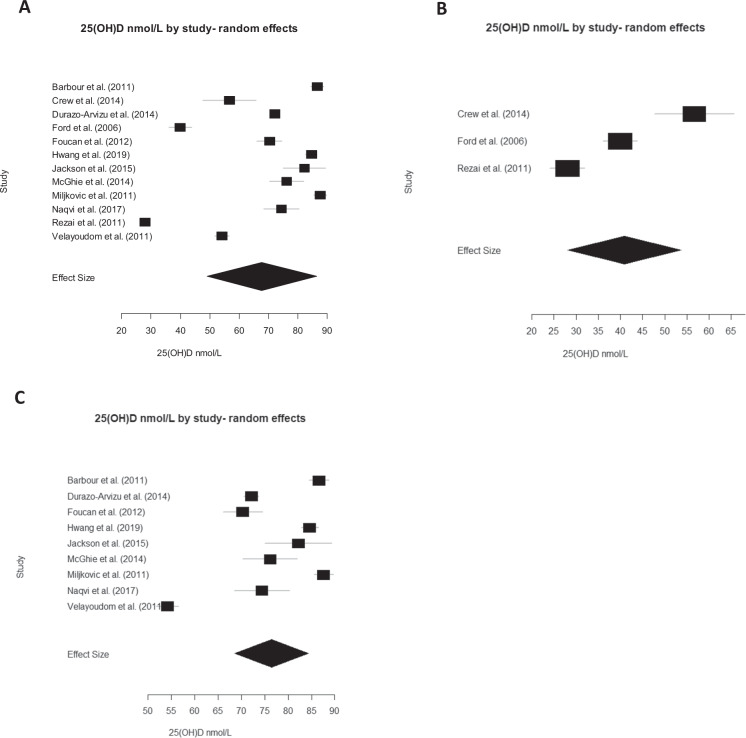
Random effects meta-analyses of 25(OH)D concentration of the African-Caribbean population. **A** All countries: summary effect = 67.8 nmol/L, 95% CI (57.9, 77.6) (*n* = 2974 participants). **B** High latitudes: summary effect = 40.9 nmol/L, 95% CI (28.1, 53.7) (*n* = 213 participants). **C** Low latitudes summary effect = 76.4 nmol/L, 95% CI (68.6, 84.3) (*n* = 2761 participants). Estimated heterogeneity for all analyses was *p* < 0.001.

A meta-analysis of 25(OH)D concentration in AfC populations living at high latitudes [27, 42–44] resulted in a pooled mean_(random)_ of 40.9 nmol/L, 95% CI (28.1, 53.7) and a pooled mean_(fixed)_ of 36.0 nmol/L, 95% CI (33.4, 38.7). At low latitudes [9, 17, 21, 33–39], a pooled mean_(random)_ of 76.4 nmol/L, 95% CI (68.6, 84.3) and a pooled mean_(fixed)_ of 77.3 nmol/L, 95% CI (76.5, 78.1) was found. Statistically significant heterogeneity was present in all models (*p* < 0.001) (see Fig. 3 for random effects models, and Supplementary File and Fig. 1 for fixed effects models).

### Vitamin D dietary intake

Five studies reported on vitamin D dietary intake (*n* = 1363 participants) [33, 41, 45–47]. The pooled mean_(random)_ effect size for vitamin D dietary intake was 3.0 µg/day, 95% CI (1.67,4.31) with statistically significant heterogeneity (*P*_(heterogeneity)_<0.001). In a fixed effect model, a pooled mean_(fixed)_ of 1.84 µg/day, 95% CI (1.75, 1.93) with statistically significant heterogeneity (*P*_(heterogeneity)_ < 0.001) was found.

**Fig. 4:**
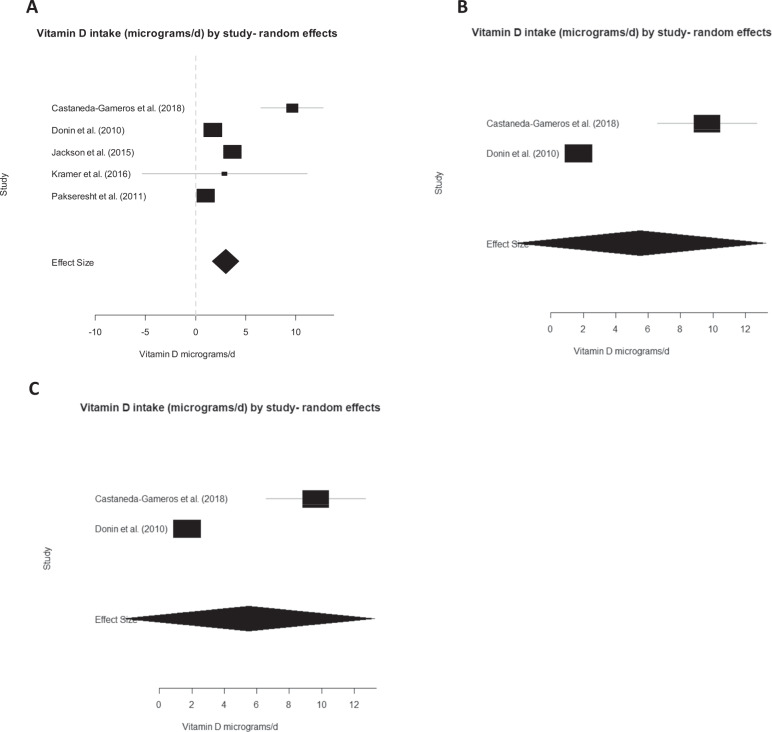
Random effects meta-analyses of vitamin D intake of the African-Caribbean population. **A** All countries: summary effect = 2.99 µg/day, 95% CI (1.67, 4.31) (*n* = 1363 participants). **B** High latitudes: summary effect = 5.51 µg/day, 95% CI (−2.26, 13.3) (*n* = 581 participants). **C** Low latitudes: summary effect = 2.38 µg/day, 95% CI (−0.112, 4.87) (*n* = 782 participants). Estimated heterogeneity for all analyses was *p* < 0.001.

For vitamin D intakes in populations living at high latitudes [46, 47], there was a pooled mean_(random)_ of 5.51 µg/day, 95% CI (−2.26, 13.3) and a pooled mean_(fixed)_ of 1.71 µg/day, 95% CI (1.61, 1.81). At low latitudes [33, 41, 45] a pooled mean_(random)_ of 2.38 µg/day, 95% CI (−0.112, 4.87) and a pooled mean_(fixed)_ of 2.68 µg/day, 95% CI (2.43, 2.93) was found. Statistically significant heterogeneity was present in all sub-group models (*p* < 0.001). A sensitivity analysis showed consistent results across all analyses (see Fig. 4 for random effects models, and Supplementary File and Fig. 2 for fixed effects models and Table 8 for the sensitivity analysis).

## Discussion

This systematic review and meta-analysis assessed the vitamin D status of AfC populations globally. A random effects meta-analysis found this population, as a whole, to have sufficient (based on our cut-offs: sufficiency >50 nmol/L [28]) mean 25(OH)D concentration at 67.8 nmol/L, yet had low mean dietary intakes of vitamin D at only 3.0 µg/day.

However, vitamin D status varied by latitude. We found higher 25(OH)D concentrations in AfC populations living at lower latitudes, compared with higher latitudes. First, using simple correlation, we found a strong inverse association between 25(OH)D status and distance from the equator. Second, random effects meta-analyses showed a mean 25(OH)D concentration at low latitudes of 76.4 nmol/L, which would be classified as sufficient (>50 nmol/L [28]). However, meta-analyses showed that populations at high latitudes had insufficient concentrations, with a mean 25(OH)D concentration of 40.9 nmol/L. Random effects meta-analyses for vitamin D intake also showed a latitude differential. For high latitudes, intakes were 5.5 µg/day. Equivalent intakes for low latitudes were 2.4 µg/day.

Of note, our sensitivity analyses showed consistent meta-analysis findings for both 25(OH)D concentration and vitamin D dietary intake, suggesting the analyses were robust.

We can speculate as to why AfC populations living at higher latitudes have poorer vitamin D status than do those at lower latitudes. Populations residing closer to the equator have an abundance of annual sunshine hours, and greater ability to synthesise vitamin D from intense UVB radiation due to the zenith angle of the sun [11, 36]. Accordingly, the studies included in this review all found vitamin D sufficiency in populations living at these low latitudes. As an exception, ‘insufficient’ 25(OH)D levels were found only in participants from two studies [36, 39]. However, vitamin D cut-offs applied in these studies were high (<75 nmol/L) and when compared to our cut-offs, these participants were sufficient (>50 nmol/L). Additionally, these participants either had T2DM or were people with chronic renal disease that were undergoing haemodialysis [36, 39], both of which are associated with vitamin D deficiency.

Our findings are similar to those from studies of other ethnic minorities living at high latitudes. For example, South Asian populations living in the UK are known to have high levels of vitamin D deficiency (<25 nmol/L) [48]. Our findings suggest that the extent of vitamin D deficiency, although higher than in WE, may not be as extensive as those seen in the South Asian population.

Vitamin D intake was low in AfC populations across all latitudes. However, when considering the different dietary recommendations by country, suboptimal intakes were more common at high latitudes, that is, the UK [22]. The highest intake of vitamin D in the UK was found in a small sub-sample of a study by Castaneda-Gameros et al. [22, 46], this may be due, in part, to some participants taking supplements that may have artificially elevated their vitamin D intakes [46]. Interestingly, those UK participants who took vitamin D and calcium supplements met the recommended nutrient intake (RNI) for both nutrients, suggesting a supplement may be needed for AfC to meet recommendations at this latitude, with its recognised lower sunlight levels [22, 46]. Closer to the equator, adherence to locally recommended dietary intakes (RDI) was achieved in two out of three studies [33, 41] but is likely attributed to lower RDI recommendations in the countries studied which take into consideration the abundance of sunshine seen in these countries [29].

The findings of this review suggest that awareness of vitamin D deficiency needs to be raised amongst AfC populations living at higher latitudes. Furthermore, vitamin D deficiency should also be of concern at lower latitudes, as although deficiency rates are lower, sufficient sun exposure may be difficult for some to achieve and others may be predisposed to deficiency due to associated chronic conditions [49, 50]. The trend for low dietary vitamin D intake has led to recommendations to consume vitamin D rich foods, fortified products, and to consider taking supplements, especially for those living at higher latitudes where there is greater risk of vitamin D deficiency [22]. This may be of importance to scientists, policy makers and clinicians working in these higher latitude countries, as very little is known in relation to the extent of deficiency, as well as strategies to address deficiency in this population group. The findings of our review highlight a need for education on the importance of vitamin D, whilst further research is needed on the impact of vitamin D deficiency in AfC populations.

Strengths of this review are that it provides a novel focus on the AfC population and illustrates vitamin D status is associated with latitude of residence. However, this review does have some limitations. Firstly, the health of the populations varied between studies, with some populations having pre-existing medical conditions that may exacerbate vitamin D deficiency. Also, all of the high latitude studies were from the UK and the US, which limits generalisability of the results. Additionally, those who self-identified as African-Caribbean, may have been of mixed race which could have impacted on their vitamin D status, due to variations in skin colour.

The methods used to analyse 25(OH)D concentration and dietary vitamin D intake also varied between studies. Some studies on black populations, which could have included AfC, may have been unnecessarily excluded due to insufficient information about participant sub-ethnicity. The use of vitamin D supplements was also not recorded in all studies, which may too have impacted on results. Converting skewed data from median and IQR to mean and SD to perform the meta-analysis may have limited the accuracy of the results. The Newcastle Ottawa Scale was not a good fit to all studies, especially for case–control studies which had to be analysed using the adapted scale. Therefore, this subjective process was used as a general guide to assess quality, rather than providing a definitive score.

## Conclusion

This systematic review and meta-analysis found sufficient vitamin D levels in AfC populations as a whole, however vitamin D insufficiency was still prevalent in AfC populations living in higher latitude countries. Dietary intake of vitamin D in AfC populations was low, globally; although populations in lower latitude countries were meeting local intake guidelines. These findings highlight a need, particularly in higher latitude countries, for public health and clinical action to improve the vitamin D awareness and status of AfC populations. This could include strategies to increase vitamin D intake, as well as the use of safe sunlight exposure, as appropriate. Further studies on the association between vitamin D and health outcomes, using larger sample sizes, is needed in this population, especially at higher latitudes.

## Supplementary information

Supplementary Files
