# Supplementary material for: Global Perspective of the Vitamin D Status of African-Caribbean Populations: A Systematic Review and Meta-analysis
Source: Eur J Clin Nutr. 2021 Jul 19;76(4):516–26. doi: 10.1038/s41430-021-00980-9 (PMC8993683; doi:10.1038/s41430-021-00980-9)
Supplement: Supplementary file 1 — Supplementary Files [file 41430_2021_980_MOESM1_ESM.docx]

# **Supplementary Files**


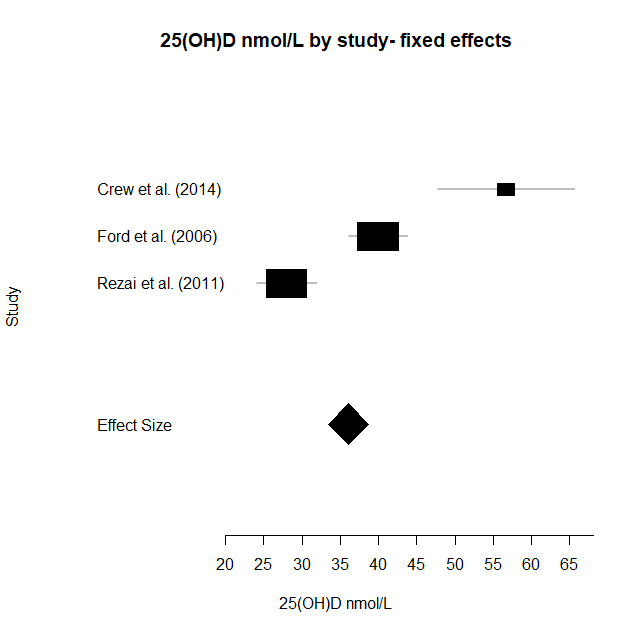


**A**  **B**


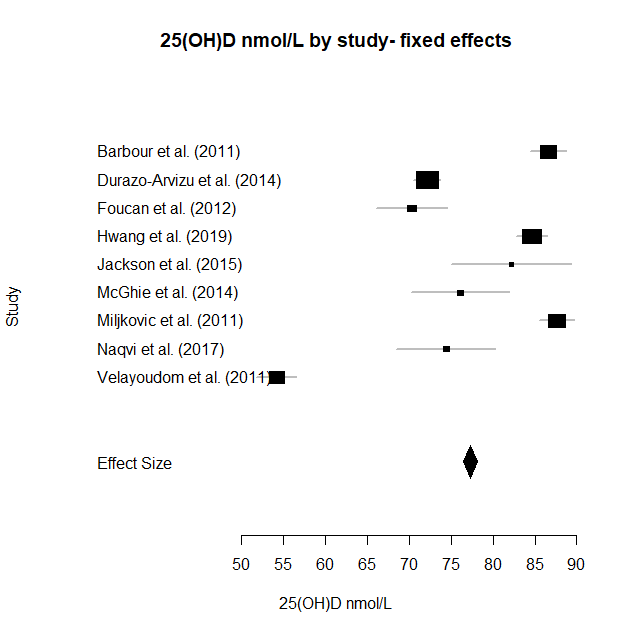


**C**

## **Figure 1:** Fixed effects meta-analyses of 25(OH)D concentration of the African-Caribbean population. **A.** All countries: Summary effect= 73.5nmol/L, 95% CI (72.7, 74.3) (n=2974 participants) **B.** High latitudes: Summary effect=36nmol/L, 95% CI (33.4, 38.7) (n=213 participants) **C.** Low latitudes Summary effect=77.3nmol/L, 95% CI (76.5, 78.1) (n=2761 participants). Estimated heterogeneity for all analyses was p<0.001.


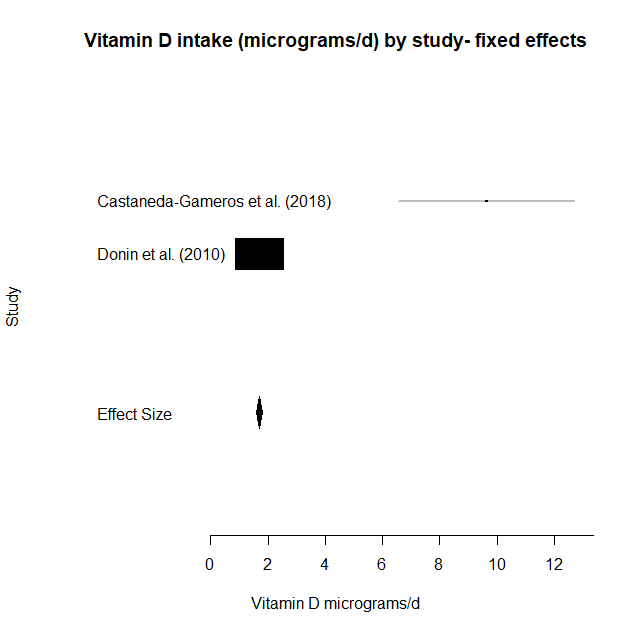


**A B**


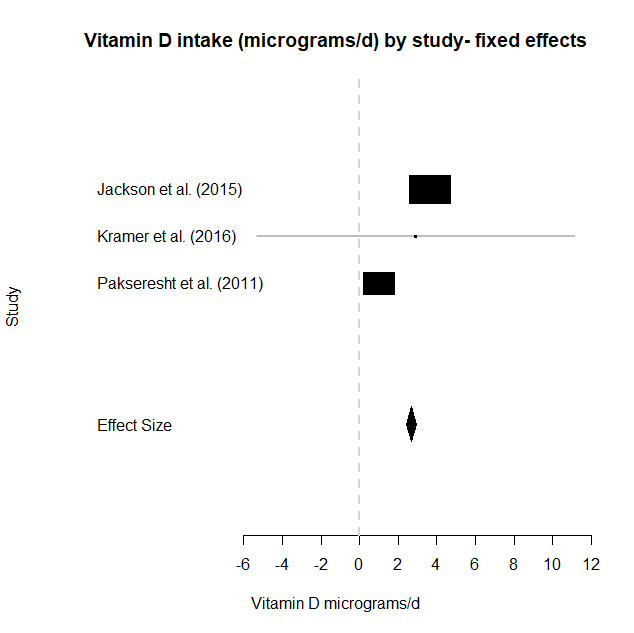


**C**

## **Figure 2:** Fixed effects meta-analyses of vitamin D intake (µg/day) of the African-Caribbean population: **A.** All countries: Summary effect= 1.84µg/day, 95% CI (1.75, 1.93) (n=1363 participants).

**B.** High latitudes: Summary effect=1.71µg/day, 95% CI (1.61, 1.81) (n=581 participants). **C.** Low latitudes: Summary effect=2.68µg/day, 95% CI (2.43,2.93) (n=782 participants). Estimated heterogeneity for all analyses was p<0.001.

## **Table 1:** Database Searches

| **Database** | **Search Terms** |
| --- | --- |
| PUBMED, Web of Science and Scopus | (Vitamin D intake OR dietary vitamin D OR vitamin D supplement OR vitamin D consumption OR Vitamin D status OR vitamin D level OR 25(OH)D OR 25-hydroxy*) AND (African-Caribbean OR afro-Caribbean OR African OR Caribbean) |
| **Database** | **Search Results** |
| PUBMED | 810 papers |
| Web of Science | 1207 papers |
| Scopus | 187 papers |

The search was conducted in October 2019. Unless otherwise stated, search terms were free text terms. Only human papers and papers written in the English language were considered.

## **Table 2:** Recommended Vitamin D cut-off ranges and sources of these values for biomarkers and diet across different latitudes included in the review

| Vitamin D status | 25(OH)D concentration (nmol/L) | Reference | Reasoning |
| --- | --- | --- | --- |
| Deficiency | <25 | Scientific Advisory Committee on Nutrition (SACN)(22) | Increased risk of poor musculoskeletal health below this level  Note: not a clinical threshold diagnosis |
| Insufficiency | >25-50 | Institute of Medicine (IOM)(28) | Inadequate for bone health and general health in healthy populations |
| Sufficiency | >50 | IOM(28) | Adequate for bone health and general health in healthy populations |

Note: 1nmol/L = 0.4ng/mL. To convert nmol/L to ng/ml, divide by 2.50

## **Table 3:** Recommended vitamin D intakes across different latitudes included in the review

| **Country** | **Recommended intake (µg/day)** | | **Reference** | **Reasoning** |
| --- | --- | --- | --- | --- |
| United Kingdom | 0 months-4 years | 8.5-10 | Scientific Advisory Committee on Nutrition (SACN) (22) | Recommended nutrient intake (RNI): The average amount needed by 97.5% of the population to maintain a serum 25(OH)D concentration ≥ 25 nmol/L when UVB sunshine exposure is minimal  Note: 0 months-4 years is a ‘safe intake’ due to insufficient data to set an RNI |
|  | >4 years | 10 |  |  |
| Caribbean | 0-3 years | 10 | Caribbean Food and Nutrition Institute (29) | Recommended dietary allowance. Allowance of vitamin D is low due to abundance of sunshine seen in Caribbean countries. A gradual transition to a lower intake with age. |
|  | 4-6 years | 5 |  |  |
|  | >7 years | 2.5 |  |  |

## **Table 4:** Reasoning for excluding papers from review

| **Stage of Screening** | **Number of papers screened against inclusion criteria** | **Reason for exclusion** | **Number of papers excluded at this stage** |
| --- | --- | --- | --- |
| Screening of titles and abstracts | 1507 | Ethnicity of population (African, South African, East African, Finnish, Saudi, Japanese, Chinese, Asian, Spanish, Indian), Hispanic (black and non-black), animal studies, plant studies, did not measure vitamin D as an outcome, review (SLR, narrative), food (e.g. content of vitamin D in milk) | 1260 papers were excluded at this stage. |
| Screening of full texts | 247 | Incorrect Population (Puerto Rican/Hispanic/Latino/other: 15, Black/African American/non-Hispanic black: 158, African/African ancestry: 18, Caribbean: 6), Population included African Caribbean, however various degrees of African heritage were not analysed separately: 7,  Ethnicity not a variable or not measured against vitamin D status: 7, did not measure vitamin D as an outcome: 4, Non English: 1, Reviews, case reports, book chapters or conference abstracts: 12 | 228 papers were excluded at this stage |
| Inclusion in systematic review | The remaining 19 studies that fit the inclusion criteria were included in this review. |  |  |

**Table 5: Included studies in the systematic review and meta-analysis**

| **Systematic review: n=19 papers** | | | |
| --- | --- | --- | --- |
|  | **Low latitudes** | **High latitudes** | **Total** |
| **25(OH)D** | n= 10  *Barbour et al. 2011*  *Chiang et al. 2017*  *Durazo-Arvizu et al. 2014*  *Foucan et al. 2012*  *Hwang et al. 2019*  *Jackson et al. 2015*  *McGhie et al. 2014*  *Miljkovic et al. 2011*  *Naqvi et al. 2017*  *Velayoudom-Cephise et al. 2011* | n=4  *Crew et al. 2014*  *Ford et al. 2006*  *Patel et al. 2013*  *Rezai et al. 2011* | 14 |
| **Dietary intake** | n=3  *Jackson et al. 2015*  *Kramer et al. 2016*  *Pakseresht et al. 2011* | n=3  *Castaneda-Gameros et al. 2018*  *Donin et al. 2010*  *Rees et al. 2005* | 6 |
|  | | | 20 *(includes Jackson et al. 2015 twice in both dietary intake and 25(OH)D)* |
| **Meta-analysis: n =16 papers** | | |  |
| **25(OH)D** | n=9  *Barbour et al. 2011*  *Durazo-Arvizu et al. 2014*  *Foucan et al. 2012*  *Hwang et al. 2019*  *Jackson et al. 2015*  *McGhie et al. 2014*  *Miljkovic et al. 2011*  *Naqvi et al. 2017*  *Velayoudom-Cephise et al. 2011* | n=3  *Crew et al. 2014*  *Ford et al. 2006*  *Rezai et al. 2011* | 12 |
| **Dietary intake** | n=3  *Jackson et al. 2015*  *Kramer et al. 2016*  *Pakseresht et al. 2011* | n=2  *Castaneda-Gameros et al. 2018*  *Donin et al. 2010* | 5 |
|  | | | 17 *(includes Jackson et al. 2015 twice in both dietary intake and 25(OH)D)* |

## **Table 6:** Newcastle Ottawa Scale quality analysis table of case control study (30)

| ***Reference*** | ***Selection*** | | | | ***Comparability*** | ***Exposure*** | | | ***Total Score*** |
| --- | --- | --- | --- | --- | --- | --- | --- | --- | --- |
|  | Is the case definition adequate | Representativeness of the cases | Selection of controls | Definition of controls | Comparability of cases and controls on the basis of the design or analysis | Ascertainment of exposure | Same method of ascertainment for cases and controls | Non-Response rate |  |
| **Jackson et al. (2015)** |  |  |  |  |  |  |  |  | *7* |

*Note: Selection category (maximum score of 4). Comparability category (maximum score of 2). Exposure category (maximum score of 3). Total maximum score of 9 (30).*

Thresholds for converting the Newcastle-Ottawa scales (good, fair, and poor):

**Good quality:** 3 or 4 stars in selection domain AND 1 or 2 stars in comparability domain AND 2 or 3 stars in outcome/exposure domain

**Fair quality:** 2 stars in selection domain AND 1 or 2 stars in comparability domain AND 2 or 3 stars in outcome/exposure domain

**Poor quality:** 0 or 1 star in selection domain OR 0 stars in comparability domain OR 0 or 1 stars in outcome/exposure domain

## **Table 7:** Adapted Newcastle Ottawa Scale quality analysis table of cross-sectional and cohort studies (30-32)

| ***Reference*** | ***Selection*** | | | | ***Comparability*** | ***Outcome*** | | ***Total Score*** |
| --- | --- | --- | --- | --- | --- | --- | --- | --- |
|  | Representativeness of the sample | Sample size | Non-respondents | Ascertainment of the exposure (risk factor) | Confounding controlled for | Assessment of outcome | Statistical test |  |
| ***Barbour et al. (2011)*** |  |  |  |  |  |  |  | *7* |
| **Castaned-Gameros et al (2018)** |  |  |  |  |  |  |  | *5* |
| **Chiang et al. (2017)** |  |  |  |  |  |  |  | *6* |
| **Crew et al. (2014)** |  |  |  |  |  |  |  | *6* |
| **Donin et al. (2010)** |  |  |  |  |  |  |  | *5* |
| **Durazo-Arvizu et al. (2014)** |  |  |  |  |  |  |  | *5* |
| **Ford et al. (2006)** |  |  |  |  |  |  |  | *5* |
| **Foucan et al. (2012)** |  |  |  |  |  |  |  | *7* |
| **Hwang et al. (2019)** |  |  |  |  |  |  |  | *8* |
| **Kramer et al. (2016)** |  |  |  |  |  |  |  | *6* |
| **McGhie et al. (2014)** |  |  |  |  |  |  |  | *7* |
| **Miljkovic et al. (2011)** |  |  |  |  |  |  |  | *7* |
| **Naqvi et al. (2017)** |  |  |  |  |  |  |  | *7* |
| **Pakseresht et al. (2011)** |  |  |  |  |  |  |  | *7* |
| **Patel et al. (2013)** |  |  |  |  |  |  |  | *8* |
| **Rees et al. (2005)** |  |  |  |  |  |  |  | *6* |
| **Rezai et al. (2011)** |  |  |  |  |  |  |  | *7* |
| **Velayoudom-Cephise et al. (2011)** |  |  |  |  |  |  |  | *7* |

*Note: Selection category (maximum score of 5). Comparability category (maximum score of 2). Exposure category (maximum score of 3). Total maximum score of 10 (30, 31).*

Thresholds for converting the Newcastle-Ottawa scales (good, fair, and poor):

**Good quality:** 3 or 4 stars in selection domain AND 1 or 2 stars in comparability domain AND 2 or 3 stars in outcome/exposure domain

**Fair quality:** 2 stars in selection domain AND 1 or 2 stars in comparability domain AND 2 or 3 stars in outcome/exposure domain

**Poor quality:** 0 or 1 star in selection domain OR 0 stars in comparability domain OR 0 or 1 stars in outcome/exposure domain

## **Table 8:** Sensitivity analysis table for 25(OH)D concentration and vitamin D dietary intake (random effects model)

| **Without author** | **Random effect size** | **P** |
| --- | --- | --- |
| Barbour et al. (2011) | 71.3 95%CI (70.4, 72.2) | <0.001 |
| Crew et al. (2014) | 73.6 95% CI (72.8, 74.4) | <0.001 |
| Durazo-Arvizu et al. (2014) | 73.9 95% CI (73, 74.8) | <0.001 |
| Ford et al. (2006) | 75 95% CI (74.2, 75.8) | <0.001 |
| Foucan et al. (2012) | 73.6 95% CI (72.8, 74.4 | <0.001 |
| Hwang et al. (2019) | 70.9 95% CI (70.1, 71.8) | <0.001 |
| Jackson et al. (2015) | 73.3 95% CI (72.5, 74.2) | <0.001 |
| McGhie et al. (2014) | 73.4 95% CI (72.6, 74.2) | <0.001 |
| Miljkovic et al. (2011) | 71.1 95% CI (70.2, 72) | <0.001 |
| Naqvi et al. (2017) | 73.4 95% CI (72.6, 74.2) | <0.001 |
| Rezai et al. (2011) | 74.4 95% CI (74.6, 76.2) | <0.001 |
| Velayoudom-Cephise et al. (2011) | 72.4 95% CI (71.5, 73.2) | <0.001 |
| Castaneda-Gameros et al. 2018) | 1.85 95% CI (1.74, 1.92) | <0.001 |
| Donin et al. (2010) | 2.73 95% CI (2.48, 2.98) | <0.001 |
| Jackson et al. (2015) | 1.67 95% CI (1.57, 1.77) | <0.001 |
| Kramer et al. (2016) | 1.84 95% CI (1.75, 1.93) | <0.001 |
| Pakseresht et al. (2011) | 1.88 95% CI (1.79, 1.98) | <0.001 |
